# Improved Multilayered (Bi,Sc)O_3_-(Pb,Ti)O_3_ Piezoelectric Energy Harvesters Based on Impedance Matching Technique

**DOI:** 10.3390/s20071958

**Published:** 2020-03-31

**Authors:** Bo Su Kim, Jae-Hoon Ji, Hong-Tae Kim, Sung-Jin Kim, Jung-Hyuk Koh

**Affiliations:** 1 School of Electrical and Electronics Engineering, Chung-Ang University, Seoul 06974, Korea; applekbb@gmail.com (B.S.K.); hoon2441@naver.com (J.-H.J.); kimht0402@naver.com (H.-T.K.); 2 College of Electrical and Computer Engineering, Chungbuk National University, Cheongju 361-763, Korea; cugatech@gmail.com

**Keywords:** (Bi,Sc)O_3_-(Pb,Ti)O_3_ ceramics, piezoelectric ceramics, impedance matching

## Abstract

As a piezoelectric material, (Bi,Sc)O_3_-(Pb,Ti)O_3_ ceramics have been tested and analyzed for sensors and energy harvester applications owing to their relatively high Curie temperature and high piezoelectric coefficient. In this work, we prepared optimized (Bi,Sc)O_3_-(Pb,Ti)O_3_ piezoelectric materials through the conventional ceramic process. To increase the output energy, a multilayered structure was proposed and designed, and to obtain the maximum output energy, impedance matching techniques were considered and tested. By varying and measuring the energy harvesting system, we confirmed that the output energies were optimized by varying the load resistance. As the load resistance increased, the output voltage became saturated. Then, we calculated the optimized output power using the electric energy formula. Consequently, we identified the highest output energy of 5.93 µW/cm^2^ at 3 MΩ for the quadruple-layer harvester and load resistor using the impedance matching system. We characterized and improved the electrical properties of the piezoelectric energy harvesters by introducing impedance matching and performing the modeling of the energy harvesting component. Modeling was conducted for the piezoelectric generator component by introducing the mechanical force dependent voltage sources and load resistors and piezoelectric capacitor connected in parallel. Moreover, the generated output voltages were simulated by introducing an impedance matching technique. This work is designed to explain the modeling of piezoelectric energy harvesters. In this model, the relationship between applied mechanical force and output energy was discussed by employing experimental results and simulation.

## 1. Introduction

Many piezoelectric materials have been employed in a wide variety of applications, including sensors and actuators in industrial use [1]. Recently, piezoelectric materials with high piezoelectric charge coefficient d_33_ and piezoelectric voltage coefficient g_33_ have been tested in renewable energy applications because of their excellent mechanical-to-electrical energy conversion properties [2]. For renewable energy harvesting, piezoelectric ceramics and polymers can be employed in the nano- and micro-scale energy generators for sensors and networks [3,4,5]. A large amount of the heretofore wasted energy from mechanical shocks can be collected, converted, and reused by employing energy harvesters [6]. As energy harvesters, piezoelectric materials are used to collect electrical energy from mechanical vibrations by converting mechanical energy to electrical energy through the piezoelectric effect [7]. This amount and efficiency of the electrical energy conversion can be increased by varying the magnitude and frequency of the mechanical forces [8]. For piezoelectric energy harvesters, electrical energy is generated when a compressive or tensile stress due to mechanical forces is applied to the surface of the piezoelectric material [9]. The duration of energy generation can be only in the nano- or micro-second range. Thus, for piezoelectric energy harvesting systems, the generated output voltage is significantly high and the generation time is extremely short. Moreover, the output of 112 volts can reach to a couple of thousand volts by increasing the thickness of the piezoelectric layers. However, it is extremely difficult to properly collect the generated energy output due to the considerably short generation time on the order of micro- and milliseconds. Therefore, it is necessary to increase the generation time and decrease the output voltage to maximize the obtained piezoelectric energy. Generally, to extend the generation time of the output voltages in the piezoelectric energy harvester, a multilayered structure of piezoelectric energy harvesters with a spring structure should be considered [10], because the generation time increases linearly with the number of stacked layers. When a force is applied from the top to the bottom electrode, then the uppermost layer in the piezoelectric system operates first, and then operated sequentially to the bottom electrode. Therefore, there is a difference time slot in the operating time. Since they have a parallel connected structure, total operation or generation times can be increased. Moreover, piezoelectric energy harvesters with the spring structure can exhibit decreased output voltage with increased generation time. This implies that the form of the generated output energy can be changed by applying the spring structure [11].

In this work, multilayered 0.36(Bi,Sc)O_3_-0.64(Pb,Ti)O_3_ (hereafter BS-PT) piezoelectric material was selected and tested for energy harvester applications owing to its high piezoelectric charge coefficient of 440 pC/N, and high Curie temperature of 394.57 °C [12]. A high piezoelectric charge coefficient and Curie temperature are important parameters for the use of piezoelectrics in electronic devices. 

It is also important to extract maximum power or energy from the energy harvester sources. Therefore, it is favorable to design an effective circuit that can collect maximum power through the load. Generally, maximum power can be delivered to the load when the reactive component in the source and resistive component in the source, and the circuit should be matched through the load resistive component. In this work, various types of load values were simulated and tested for the piezoelectric energy harvesters to maximize the generated output energy.

## 2. Materials and Methods

In this study, we performed a comprehensive impedance matching analysis study on the multilayered BS-PT piezoelectric energy harvesters to obtain the maximum output energy. BS-PT stoichiometric powders were prepared by the traditional solid sintering process. Bi_2_O_3_, Sc_2_O_3_, PbO, and TiO_2_ raw powders with 99.9% purity were prepared and used as the starting materials. The powders were ball-milled for 24 h in ethyl alcohol with ZrO_2_ balls, oven-dried at 120 °C, and then calcined at 780 °C for 2 h. The heating and cooling rates were 5 °C/min. The calcined powders were mixed with 5 wt% poly vinyl alcohol and then uniaxially pressed into disks with a diameter of 20 mm under 300 MPa. These disks were sintered at 1200 °C for 2 h. The sintered BS-PT specimens were poled under a DC electric field of 4 kV/mm in a silicon oil bath at 80 °C for 30 min. Finally, piezoelectric multilayer structures were fabricated and prepared for the analysis. 

The crystalline structures of the BS-PT sintered samples were investigated by X-ray diffraction (XRD) analysis (Bruker-AXS/New D8-Advance, Germany). The surface microstructure was observed by field emission scanning electron microscopy (FE-SEM) (Carl Zeiss/SIGMA, Germany), and the capacitance of the BS-PT specimens was measured using an LCR meter (Agilent 4284A Precision, USA). The values of the piezoelectric charge coefficient d_33_ were measured using a Berlincourt quasi-static d_33_ meter (YE 2730A), and the polarization versus electric field (P-E) hysteresis loops were measured using a modified Sawyer-Tower circuit at 0.1 Hz. Mechanical force vibrators were applied to the surface of the single-, double-, triple-, and quadruple-layer BS-PT specimens connected to the reactive and resistive components. In this experiment, piezoelectric energy sources with a mechanical force vibrator connected to the resistive component, reactive component, and load were used to collect the generated output energy. To extract the maximum output electrical energies, various loads were tested and analyzed. 

## 3. Results

### 3.1. Background of 0.36(Bi,Sc)O_3_-0.64(Pb,Ti) Ceramics

**Figure 1:**
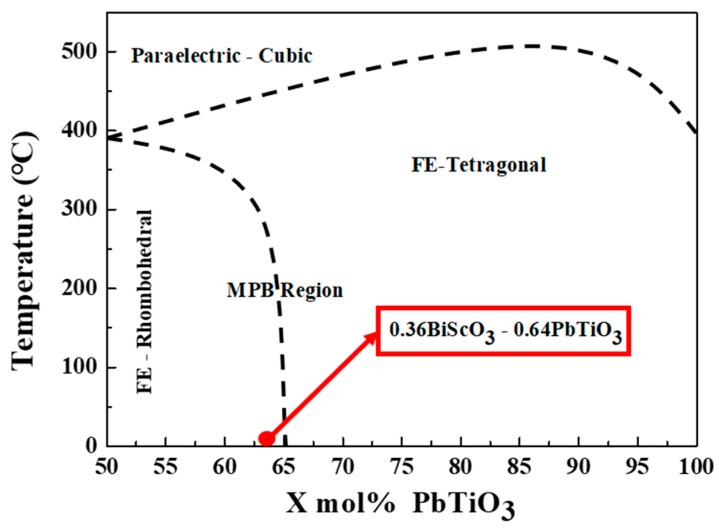
Phase diagram for the (1-*x*)(Bi,Sc)O_3_-*x*(Pb,Ti)O_3_ perovskite system [3].

Figure 1 illustrates the phase diagram for the ferroelectric (1-*x*)(Bi,Sc)O_3_-*x*(Pb,Ti)O_3_ system with a morphotropic phase boundary (MPB) observed in the vicinity of 64% PbTiO_3_ content. In a previous study, the MPB region, particularly in the rhombohedral MPB region exhibited the highest piezoelectric properties in comparison with other compositions, and therefore, in this work, the 0.36(Bi,Sc)O_3_-0.64(Pb,Ti)O_3_ ferroelectric ceramics were selected for examination, and detailed experiments were conducted to investigate the optimized calcination and sintering conditions for this composition. 

**Figure 2:**
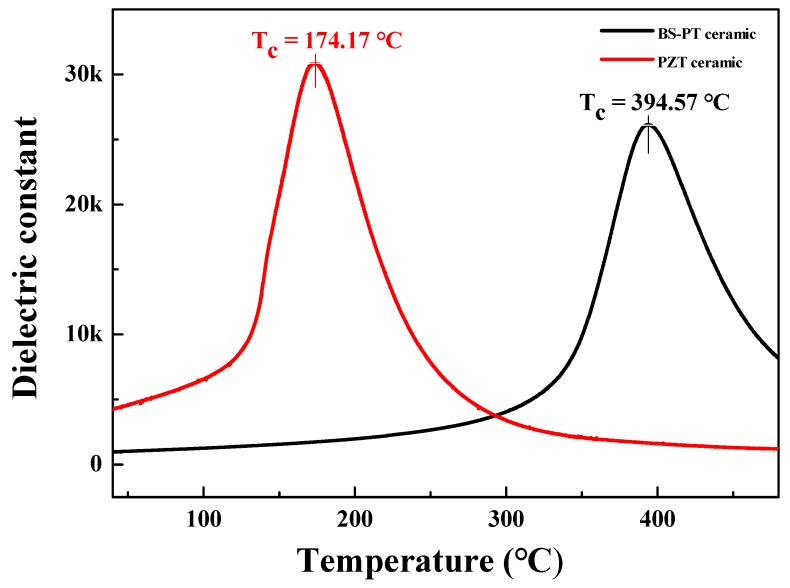
Temperature-dependent relative dielectric constant $\varepsilon_{r}$ of 0.36(Bi,Sc)O_3_-0.64(Pb,Ti)O_3_ and Pb(Zr_1-_xTix)O_3_ (PZT) ceramics.

Figure 2 depicts the temperature-dependent dielectric constant $\varepsilon_{r}$ of the BS-PT ceramic sample sintered at 1200 °C. The BS-PT ceramic exhibited a significantly high Curie temperature of 394.57 °C. In comparison with the standard Pb(Zr_1-_xTix)O_3_ (PZT) materials, BS-PT ceramics exhibited an approximately 220 °C higher Curie temperature with a higher dielectric constant. This implies that the BS-PT ceramics can be used in more harsh environmental conditions than the PZT ceramics. Figure 2 depicts the results obtained for the Mn-doped Pb(Zr_0.58_Ti_0.42_)O_3_ ceramic. Mn is added to PZT to lower the sintering temperature. Generally, PZT materials exhibit Curie temperatures between 170 °C and 370 °C [13]. Pb(Zr_0.58_Ti_0.42_)O_3_ is frequently used because the piezoelectric charge constant tends to decrease with increasing Curie temperature [14,15]. Nevertheless, a higher Curie temperature is highly favorable for the use of piezoelectric materials in industrial applications. As a representative piezoelectric material PZT-5A can be employed for the electronic device applications including energy harvesters, however, BS-PT also have some merits in applications, Both of BS-PT and PZT-5A ceramics have similar Curie temperature Tc and piezoelectric charge coefficient d33, however there are strong merits in the lower lead contents for BS-PT ceramics compared with PZT-5A ceramics. In addition, with lower dielectric permittivity of BS-PT, ceramics can help to have more higher figure of merits (FOM= d_33_ ∙ g_33_) for energy harvester applications. Here, we have summarized the representative piezoelectric properties. As shown in the Table 1, BS-PT ceramics have relatively higher piezoelectric charge coefficient with higher Curie temperature and lower dielectric constant.

### 3.2. Fabrication and Validation of 0.36(Bi,Sc)O_3_-0.64(Pb,Ti) Ceramics

**Figure 3:**
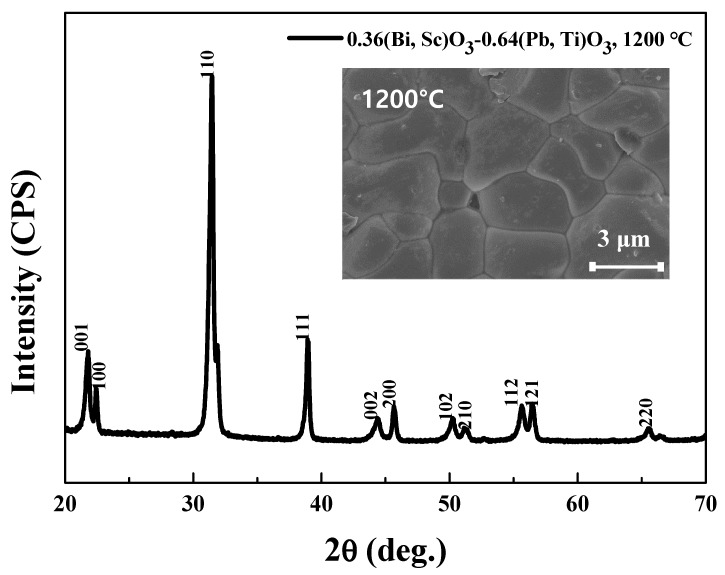
X-ray diffraction pattern data for the BS-PT piezoelectric ceramics sintered at 1200 °C. Inset shows the field emission scanning electron microscopy (FE-SEM) images of the BS-PT piezoelectric ceramics.

Figure 3 illustrates the XRD pattern data for the BS-PT piezoelectric ceramics sintered at 1200 °C. As depicted in Figure 3, the (*h00*) and (*00l*) reflection peaks are clearly observed, implying that BS-PT piezoelectric ceramics exhibit a tetragonal structure. Owing to their high tetragonality, the BS-PT ceramics are expected to exhibit high piezoelectric performance. Additionally, no peaks for the pyrochlore phase peaks were observed in the XRD pattern. Using Equation (1) and the measured 2θ values of the (*h*00) and (00*l*) peaks, the *c* and *a* lattice parameters can be calculated and analyzed according to the Nelson-Riley formula. Therefore, the film lattice parameter *c*_0_ was determined by plotting *c*_00*l*_ versus the Nelson-Riley function [16].

(1) $$\frac{c_{cos\theta }-c_{0}}{c_{0}}=A\cdot cos^{2}\theta \left(\frac{1}{sin\theta }+\frac{1}{\theta }\right)$$

where, *c*_cos_*_θ_* is an interplane distance calculated from the apparent 2θ Bragg peak position, and *A* is a fitting coefficient. The calculated *c* and *a* lattice parameters were 4.09 and 3.98 Å, respectively. The inset shows the field emission scanning electron microscopy (FE-SEM) images of the BS-PT piezoelectric ceramics. The specimen was sintered at 1200 °C for 2 h after first undergoing thermal etching. As depicted in the figure, the average grain size is approximately 3.2 μm, and the grains exhibit an irregular shape but a dense structure. 

**Figure 4:**
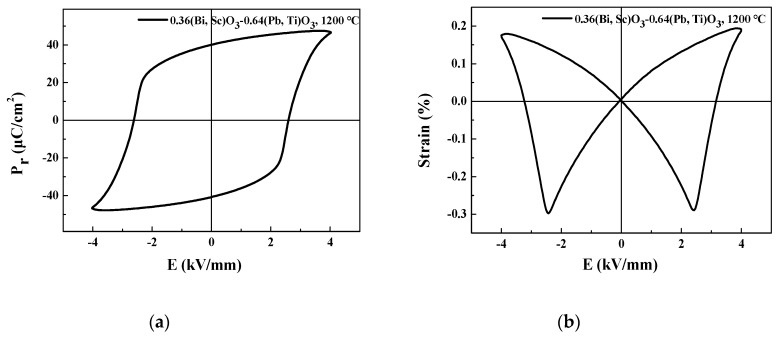
(**a**) Polarization versus electric field (P-E) hysteresis loops of the BS-PT piezoelectric ceramics at the sintering temperature of 1200 °C. (**b**) Electric field dependent strain (S-E) curves for the BS-PT piezoelectric ceramics.

Figure 4a depicts the electric field dependent polarization (P-E) hysteresis loops of the BS-PT piezoelectric ceramics sintered at 1200 °C. As observed from the figure, BS-PT ceramics exhibit an open P-E hysteresis loop with a range of 0.1 Hz. This open P-E hysteresis loop implies that the specimens require the use of a relatively high coercive electric field for removing the remnant polarization. In particular, the present specimen exhibits a high remnant polarization of 40.2 μC/cm^2^. The external voltage applied to the specimen reaches ± 4 kV/mm. These high coercive electric field and remnant polarization values were similar to the values reported in the literature [17]. This open P-E hysteresis observed for the BS-PT ceramics can be compared with the P-E loops of PZT-based materials that exhibit high piezoelectric performance for device applications [18]. Materials with open P-E hysteresis loops with high coercive electric field and remnant polarization values can be used in piezoelectric passive damper applications, because a large area of the P-E hysteresis loop implies that a large amount of energy is accumulated under the application of a mechanical shock. Figure 4b depicts the electric field dependent strain (S-E) curves for the BS-PT piezoelectric ceramics. As observed from the figure, the specimen exhibits non-linear S-E hysteresis loops. At an applied electric field of approximately 4 kV/mm, the strain values approaches 0.19%, whereas for an applied electric field of approximately 1 kV/mm, the strain of the BS-PT piezoelectric ceramic is 0.07%. This large strain value is comparable with those of the PZT-based materials. For example, Pb(Zr_0.6_Ti_0.4_) exhibits the maximum strain of 0.13% [19]. Generally, in the mechanical-to-electrical energy conversion, some energy is dissipated as heat, raising the temperature of the material and degrading the piezoelectric performance. However, the BS-PT materials investigated in this work exhibit a high Curie temperature of 394.57 °C; because of this high Curie temperature, these ceramics are insensitive to the temperature variation due to the heating caused by energy dissipation, making them attractive materials for the passive damper system. The S-E hysteresis loops demonstrate that the BS-PT piezoelectric ceramics exhibit high strain values relative to other comparable piezoelectric materials.

### 3.3. Multilayer 0.36(Bi,Sc)O_3_-0.64(Pb,Ti) Piezoelctric Energy Harvester Setup

**Figure 5:**
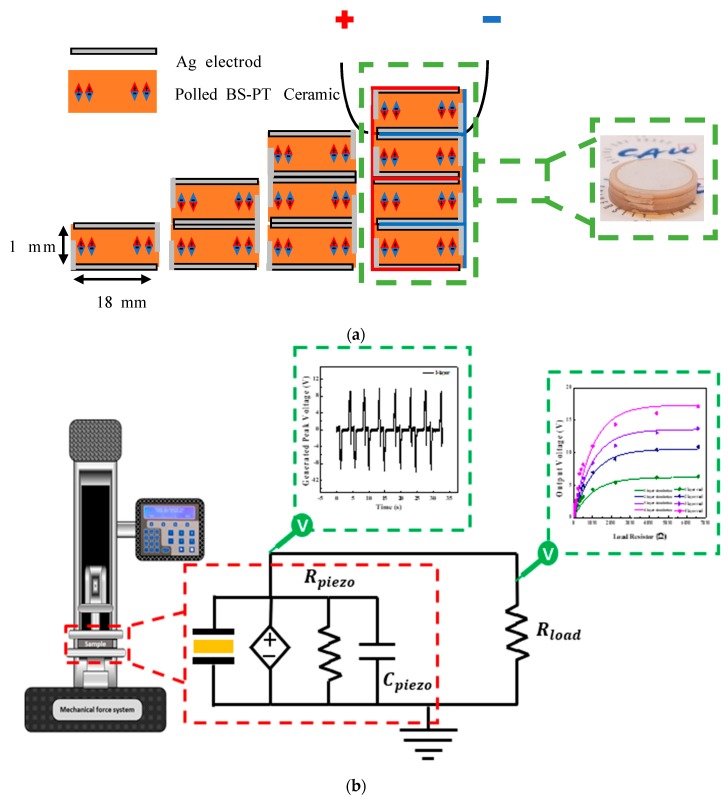
(**a**) Schematic of the 1–4 layer structured piezoelectric energy harvesters based on BS-PT multilayered ceramics. (**b**) Equivalent circuit of the piezoelectric energy harvester and the generated voltage measurement system at the load resistor using the mechanical force vibrator.

Figure 5a illustrates a schematic of the 1–4 layered structured piezoelectric energy harvesters based on BS-PT multilayered ceramics. The internal electrodes of the BS-PT multilayered ceramics are connected in parallel, as depicted in Figure 5a. To increase the generated output energy, a capacitor structure connected in parallel is designed and fabricated. Since piezoelectric ceramics are connected in the parallel structure, the output voltages are generated for a relatively long time in comparison with that generated using a single-piezoelectric structure. As a result of this extended output voltage generation time, multilayered BS-PT piezoelectric materials can generate high levels of energy. Multilayered piezoelectric devices must be connected in parallel. In the circuit, top electrode generated positive current, and the bottom electrode generate negative current, respectively. If the multilayered piezoelectric devices are connected in series, then the bottom electrode of the 1st layer and top electrode of the 2nd layer are facing each other. Therefore, the positive current in the 1st layer and negative current in the 2nd layer should be canceled. As a result, the output energy can be reduced. Figure 5b depicts the designed output energy measurement system incorporating a load resistor. A mechanical force of 500 N was applied to the piezoelectric energy harvester at a frequency of 0.2 Hz. The piezoelectric energy harvester can be described using an equivalent circuit with the mechanical force dependent voltage sources, internal resistor R_piezo_, and capacitive component C_piezo_ connected in parallel. As depicted in the figure, the voltage across the load resistor can be increased because the generated piezoelectric charges are accumulated in the load capacitors. 

**Figure 6:**
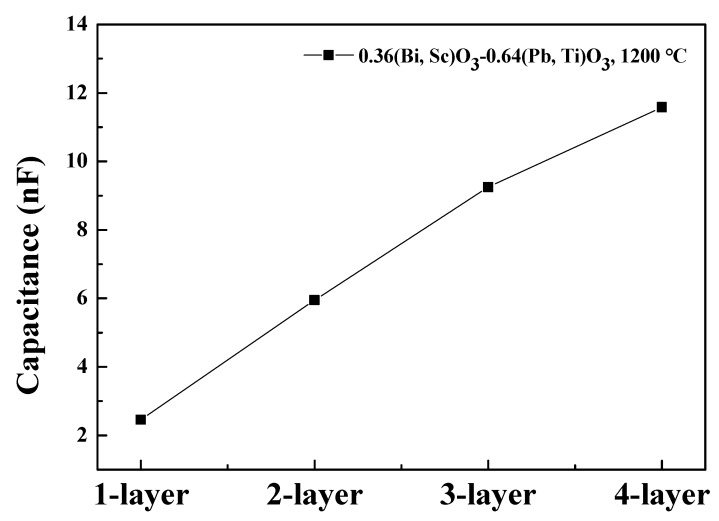
Measured capacitance of the 1–4 layer structured piezoelectric energy harvesters based on the BS-PT multilayered energy harvester.

Figure 6 depicts the stored and measured capacitances of the 1–4 layer parallel connected capacitive structures for piezoelectric energy harvesters based on the BS-PT multilayered ceramics as a function of the number of layers. It is observed that the stored capacitance increases with the increasing number of layers because the internal electrodes of the BS-PT multilayered ceramics are connected in parallel. The capacitance values of the single, double, triple, quadruple layers were 2.46, 5.95, 9.25, and 11.58 nF, respectively, and the capacitance increased proportionally with the number of layers. This implies that the multilayered piezoelectric energy harvesters were successfully connected in the parallel structure. Therefore, we can expect that the output energy can be linearly increased by increasing the number of layers [20].

### 3.4. Output Energy of the Multilayer 0.36(Bi,Sc)O_3_-0.64(Pb,Ti) Piezoelectric Energy Harvester 

**Figure 7:**
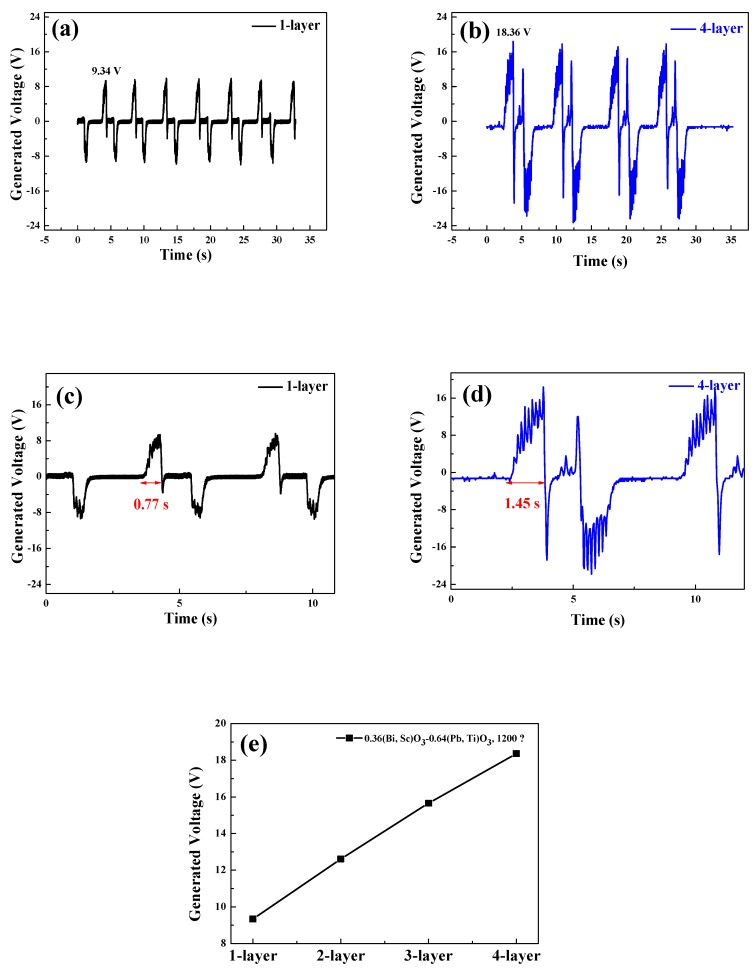
Generated voltage and duration time for single- and quadruple-layered piezoelectric BS-PT energy harvesters with a frequency of 0.2 Hz (**a**–**d**) show a detailed figure of (a) and (b) to show elongated generation time more clearly for the single- and quadruple-layered BS-PT energy harvesters with a frequency of 0.2 Hz. (**e**) Peak voltage measurements for the 1–4 layer BS-PT piezoelectric energy harvesters.

Figure 7a,b depicts the generated voltages of the piezoelectric energy harvesters as a function of time. This energy harvester is connected with single- and multi-layered BS-PT ceramics. A mechanical force of 500 N was applied to the piezoelectric energy harvesters at a frequency of 0.2 Hz, corresponding to a human with a weight of 50 kg walking on top of the piezoelectric energy harvesters. The positive and negative output voltages were generated when the compressive and tensile forces were applied to the piezoelectric energy harvesters. It was observed that forces of equal magnitude were applied to the devices. In every experiment, we found this small peak with applied high mechanical force condition. We believe this small peak probably come from the recovery of piezoelectric materials after removing the mechanical forces. After removing the high positive forces, then the piezoelectric material experiences a strong recovery negative force to restore the shape. Therefore, small negative peaks are always observed. Figure 7c,d depicts the generation time of output voltage in the single- and multi-layered piezoelectric energy harvesters as a function of time. The measured generation time of output voltages were 0.77 and 1.45 s for the single- and quadruple-layer structure piezoelectric devices, respectively. This result shows that the output voltage generation time increases as the number of layers increases. Figure 7e depicts the generated peak voltages of the 1–4 multilayered BS-PT piezoelectric ceramics with 1–4 layers measured at the load resistor. The measured generated voltages of specimens were 9.34, 12.61, 15.66, and 18.36 V for the single-, double-, triple-, and quadruple-layer structure piezoelectric energy harvesters, respectively. As shown in the Table 2, we check the generated peak voltage for the 1–4 layer BS-PT piezoelectric energy harvesters in 500 N.

Since they were connected in parallel, the generated output voltage increased with increasing number of layers. Therefore, a voltage similar to that obtained using PZT-based materials was generated [21]. 

**Figure 8:**
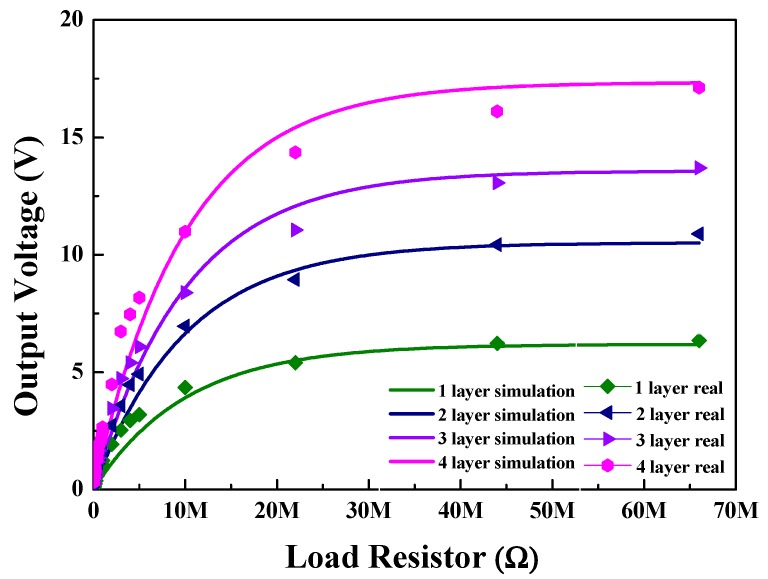
Generated output voltages of multilayered BS-PT piezoelectric ceramics obtained by varying the load resistors under the application of a mechanical force of 500 N.

Figure 8 depicts the generated output voltages of multilayered BS-PT piezoelectric ceramics obtained by varying the load of the resistor. As observed from the figure, the output voltage increased with increasing load of the resistor. Since the load resistor is connected to the piezoelectric energy harvester in series, the generated output voltage can be increased with a contribution of the internal resistive component. Therefore, as the load resistor value increased, the resistive component contribution in the load part increased as well. As a result, the output voltages should be increased. Using Equation (2), the relation between a resistive load and the output voltage can be expressed as follows.

(2) $$V_{o}=V_{p}\left(1-e^{-\frac{R_{L}}{R_{int}}}\right),$$

where V_o_ is the generated output voltage applied to the load resistor, V_p_ is the generated peak voltage, R_L_ is the load resistor, and R_int_ is the internal resistance related to the saturated output voltage. 

There are some theoretical papers, in which simulation and modeling for piezoelectric energy harvesters were employed to explain the energy converting behavior [22,23]. However, there are some differences that can be found. In the Junior et al. [22] case, the authors employed a finite element model to analyze the cantilever type resonator for energy harvesters. In this analysis, they employed thermodynamics based on Hamilton’s principle for electrostatic body, which is mainly based on the mechanical and electrical energy without magnetic effects. However, in our case, we are mainly focused on the mainly input mechanical force dependent electrical energy and their configured electric load circuit. Therefore, in a practical point of view, we are more focused on the mechanical force dependent generation energy. For the Beeby et al. [23] case, the authors employed resonant type piezoelectric generator for the energy harvester applications. They analyzed applied mechanical energies and converted energies by employing differential equation for the inertial mass system in resonators. However, in our case, we are more focused on the applied mechanical force dependent electrical input energies and system efficiencies to obtain maximum energies which we can obtain by optimizing the load circuit. The origin of Equation (2) is as follows. Considering the internal resistance of the piezoelectric element and the voltage distribution according to the internal resistance, the output voltage increases with increasing load resistance, but tends to saturate when approaching a steady state. As depicted in Figure 8, the fitting to this equation was conducted using the iterative least mean square error minimization method. The fit values and measured values were well-matched with each other. It appears that our assumption of internal resistance related generated output voltages were related with each other. In our previous study [23], we measured the generated output energy of a PZT-based piezoelectric energy harvester, wherein tape-casting-based multilayered structures were employed and tested. As we employed a multilayered structure, the capacitance values approached the μF level. As a result, the load impedance exhibited different values. However, the generated output voltages exhibited similar values. In this experiment as well, the generated output voltages increased with the load resistor value and then become saturated. Based on Figure 8, the maximum generated output energy can be estimated based on the saturated output voltage values [24].

**Figure 9:**
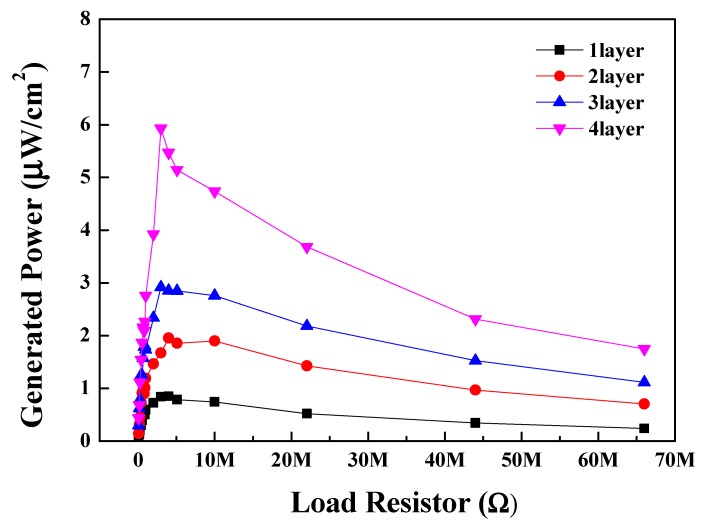
Generated power of the multilayered BS-PT piezoelectric energy harvesters depending on the load resistors for the mechanical force of 500 N.

Figure 9 depicts the generated output power of the multilayered BS-PT piezoelectric energy harvesters as a function of the resistor load. In contrast to Figure 8, which depicts the generated output voltages, the generated output power can exhibit maximum values when the load resistor has similar internal resistance values. According to the circuit theory, an external matching circuit should have similar impedance values to the internal system; otherwise, the maximum output power cannot be obtained. To obtain the maximum power, the derivative of the equation for the total system should be considered. As a result, Equations (3) and (4) were obtained. Therefore, the external load resistor R_L_ should have similar values to the internal resistor value R_t_. The red dashed line in the energy harvester circuit shown in Figure 5b represents the equivalent circuit of the piezoelectric harvesters. In the equivalent circuit, R_piezo_ and C_piezo_ are the internal resistance and capacitance of the piezoelectric energy harvester, respectively. Based on the impedance matching theory, when the load resistor R_load_ has similar values to R_piezo_, the generated output energy can be maximized. Using Equation (3), the relation between the resistive load and the generated power can be expressed as

(3) $$P=\frac{{\left|V_{o}\right|}^{2}R_{L}}{{\left(R_{t}+R_{L}\right)}^{2}}$$

(4) $$R_{piezo}=R_{L},\;\;\;\frac{dP_{max}}{dR_{L}}=0$$

where V_o_ is the generated output voltage, R_L_ is the load resistance, and R_piezo_ is the internal resistance of the energy harvesters. Using Equation (4), the maximum values of generated power can be obtained when the resistive part in the load component has the same value as that of the piezoelectric ceramic materials and other circuit components. As the load resistive component approaches 3 MΩ, the piezoelectric energy harvester system exhibits maximum power values of 5.93 μW/cm^2^ for the quadruple-layer system, demonstrating high performance similar to that of the PZT-based energy harvester [23,24]. The load resistor values for the maximum power of energy generation decreased with increasing number of layers. Due to the parallel connection of the piezoelectric component layers, the resistive component value decreased with increasing number of layers. As shown in the Table 3, we check the matching impedence and generated power for the 1–4 layer BS-PT piezoelectric energy harvesters in 500 N.

## 4. Conclusions

In this study, we analyzed 0.36(Bi,Sc)O_3_-0.64(Pb,Ti)O_3_ piezoelectric ceramics and designed a multilayer piezoelectric energy harvester. The ceramics exhibited a high piezoelectric charge coefficient of 440 pC/N, a high Curie temperature value of 394.57 °C, and a tetragonal structure as observed by XRD. The calculated c and a lattice parameters were 4.09 and 3.98 Å, respectively, based on the Nelson-Riley function. An examination of the FE-SEM image revealed that the surface of the BS-PT ceramics exhibited a uniform grain distribution, with the average grain size of approximately 3.2 µm. We successfully manufactured piezoelectric energy harvesters based on the BS-PT ceramics. The generated peak voltages of the single-, double-, triple-, and quadruple-layer BS-PT ceramics were 9.34, 12.61, 15.66, and 18.36 V, respectively, under the application of a mechanical force of 500 N. Therefore, the output power values were 0.85, 1.95, 2.92, and 5.93 μW/cm^2^ for the single, double, triple, and quadruple layers, respectively, with the optimized load resistor. In this work, impedance matching and modeling of energy harvesting were conducted. Modeling was conducted for the piezoelectric generator component by introducing the mechanical force dependent voltage sources and load resistors and piezoelectric capacitor connected in parallel, and the generated output voltages were simulated by introducing the impedance matching technique. We obtained the relationship between the load resistance and the output voltage in the circuit we tested and proved it through Equation (2). This work is designed to explain the modeling of piezoelectric energy harvesters. In this model, the relationship between applied mechanical force and output energy was discussed by employing experimental results and simulation.

## Figures and Tables

**Table 1:** Characteristics of the prepared 0.36(Bi,Sc)O_3_-0.64(Pb,Ti)O_3_ (BS-PT).

Materials	Piezoelectric Constant (*d_33_*)	Dielectric Constant (*Ɛ_r_*)	Curie Temperature (*T_c_*)	Bulk Density (*g/cm^3^*)
0.36(Bi,Sc)O_3_-0.64(Pb,Ti)O_3_[12]	440 pC/N	1479	394.57 °C	7.44
Mn dopedPb(Zr_0.58_Ti_0.42_)O_3_	581 pC/N	1577	174.17 °C	7.2
Pb(Zr_0.52_Ti_0.48_)O_3_[11]	223 pC/N	1240	377 °C	7.55
CeramTec PZT-5A [23]	374 pC/N	1700	370 °C	7.75

**Table 2:** Generated peak voltage for the 1–4 layer BS-PT piezoelectric energy harvesters.

Layer	Input Force	Voltage
1-layer	500 N	9.34 V
2-layer	500 N	12.61 V
3-layer	500 N	15.66 V
4-layer	500 N	18.36 V

**Table 3:** Generated power of the 1–4 layer BS-PT piezoelectric energy harvesters.

Layer	Matching Impedence	Generated Power
1-layer	4 MΩ	0.85 μW/cm^2^
2-layer	4 MΩ	1.95 μW/cm^2^
3-layer	3 MΩ	2.92 μW/cm^2^
4-layer	3 MΩ	5.93 μW/cm^2^
